# Combining long-lasting insecticidal nets and indoor residual spraying for malaria prevention in Ethiopia: study protocol for a cluster randomized controlled trial

**DOI:** 10.1186/1475-2875-13-S1-P25

**Published:** 2014-09-22

**Authors:** Wakgari Deressa, Eskindir Loha, Meshesha Balkew, Alemayehu Desalegne, Taye Gari, Teshome Gebremichael, Oljira Kenea, Daddi Jima, Bjarne Robberstad, Hans J Overgaard, Bernt Lindtjørn

**Affiliations:** 1 Addis Ababa University, Addis Ababa, Ethiopia; 2 Hawassa University, Hawassa, Ethiopia; 3 Ethiopian Public Health Institute, Addis Ababa, Ethiopia; 4 University of Bergen, Bergen, Norway; 5 Norwegian University of Life Sciences, Ås, Norway

## 

Long-lasting insecticidal nets (LLINs) and indoor residual spraying (IRS) are the two main malaria prevention strategies in Ethiopia. Although both interventions have been shown to be effective in reducing malaria transmission when applied independently, there is conflicting evidence that the two in combination is better than either one alone. The main objectives of this trial are to determine the added protection value against malaria and to evaluate the cost-effectiveness when applying IRS and LLINs together, or LLINs or IRS independently.

This trial will be conducted in Adami Tullu district of Oromia Regional State in Ethiopia from 2014 to 2016. The project will use a cluster randomized controlled trial, with four “arms”: IRS+LLINs, LLINs alone, IRS alone and control (routine practice). The sample size includes 40 clusters in each arm, each cluster with 35-45 households. Each household and each inhabitant in the household will be given a unique identification number. Households will be mapped using global positioning system. At the start of the trial, all households in the IRS+LLINs and LLINs alone “arms” of the study will be provided new LLINs free of charge. IRS with an insecticide (propoxur) will be applied in IRS+LLINs and IRS alone arms twice a year throughout the study.

Each household will be visited weekly, and blood samples will be collected from each household member with fever or history of fever. Thick and thin blood smears will be taken by finger prick and rapid diagnostic tests will be used to detect malaria at field level. Data on all self-reporting malaria patients attending health posts will be collected. The cost-effectiveness and entomological studies will be simultaneously conducted. Analysis will be based on intention to treat principle. Ethical clearance was obtained from the Ministry of Science and Technology in Ethiopia and the University of Bergen.

This trial aims to provide evidence on the combined use of IRS and LLINs for malaria prevention. We aim to answer the following research questions: Can the combined use of LLINs and IRS significantly reduce malaria incidence compared with the use of LLIN or IRS alone? And is the reduced incidence justifiably compared to the added costs? Will the combined use of LLINs and IRS reduce vector density, infection, longevity and the entomological inoculation rate? Such data is crucial in order to maximize the impact of the intervention on malaria morbidity and mortality.

